# Long-term outdoor air pollution and DNA methylation in circulating monocytes: results from the Multi-Ethnic Study of Atherosclerosis (MESA)

**DOI:** 10.1186/s12940-016-0202-4

**Published:** 2016-12-01

**Authors:** Gloria C. Chi, Yongmei Liu, James W. MacDonald, R. Graham Barr, Kathleen M. Donohue, Mark D. Hensley, Lifang Hou, Charles E. McCall, Lindsay M. Reynolds, David S. Siscovick, Joel D. Kaufman

**Affiliations:** 1 Department of Epidemiology, School of Public Health, University of Washington, 1959 NE Pacific St, Box 357236, Seattle, WA 98195 USA; 2 Department of Epidemiology & Prevention, Division of Public Health Sciences, Wake Forest School of Medicine, Winston-Salem, NC USA; 3 Department of Environmental and Occupational Health Sciences, School of Public Health, University of Washington, Seattle, WA USA; 4 Division of General Medicine, Mailman School of Public Health, Columbia University, New York, NY USA; 5 Division of Pulmonary, Allergy & Critical Care, Columbia University Medical Center, New York, NY USA; 6 Department of Preventive Medicine, Division of Cancer Epidemiology and Prevention, Feinberg School of Medicine, Northwestern University, Chicago, IL USA; 7 Section on Molecular Medicine, Wake Forest School of Medicine, Winston-Salem, NC USA; 8 New York Academy of Medicine, New York, NY USA

**Keywords:** Air pollution, PM_2.5_, NO_X_, ANKHD1, LGALS2, ANKRD11, BAZ2B, PPIE, DNA methylation

## Abstract

**Background:**

DNA methylation may mediate effects of air pollution on cardiovascular disease. The association between long-term air pollution exposure and DNA methylation in monocytes, which are central to atherosclerosis, has not been studied. We investigated the association between long-term ambient air pollution exposure and DNA methylation (candidate sites and global) in monocytes of adults (aged ≥55).

**Methods:**

One-year average ambient fine particulate matter (PM_2.5_) and oxides of nitrogen (NO_X_) concentrations were predicted at participants’ (*n* = 1,207) addresses using spatiotemporal models. We assessed DNA methylation in circulating monocytes at 1) 2,713 CpG sites associated with mRNA expression of nearby genes and 2) probes mapping to Alu and LINE-1 repetitive elements (surrogates for global DNA methylation) using Illumina’s Infinium HumanMethylation450 BeadChip. We used linear regression models adjusted for demographics, smoking, physical activity, socioeconomic status, methyl-nutrients, and technical variables. For significant air pollution-associated methylation sites, we also assessed the association between expression of gene transcripts previously associated with these CpG sites and air pollution.

**Results:**

At a false discovery rate of 0.05, five candidate CpGs (cg20455854, cg07855639, cg07598385, cg17360854, and cg23599683) had methylation significantly associated with PM_2.5_ and none were associated with NO_X_. Cg20455854 had the smallest p-value for the association with PM_2.5_ (*p* = 2.77 × 10^−5^). mRNA expression profiles of genes near three of the PM_2.5_-associated CpGs (*ANKHD1*, *LGALS2*, and *ANKRD11*) were also significantly associated with PM_2.5_ exposure. Alu and LINE-1 methylation were not associated with long-term air pollution exposure.

**Conclusions:**

We observed novel associations between long-term ambient air pollution exposure and site-specific DNA methylation, but not global DNA methylation, in purified monocytes of a multi-ethnic adult population. Epigenetic markers may provide insights into mechanisms underlying environmental factors in complex diseases like atherosclerosis.

**Electronic supplementary material:**

The online version of this article (doi:10.1186/s12940-016-0202-4) contains supplementary material, which is available to authorized users.

## Background

Ambient air pollution contributed to over 3.2 million premature deaths and over 76 million years of healthy life lost globally in 2010 and ranked in the top ten leading risk factors for global mortality and disease [1]. Exposure to fine particulate matter (PM_2.5_) has been associated with a variety of conditions including cardiopulmonary disease, diabetes, premature birth, low birthweight, cancer, and cognitive changes [2].

Many prior studies focused on the effect of air pollution exposure on the risk of cardiovascular mortality and morbidity. Landmark studies including the Harvard Six Cities and the American Cancer Society studies demonstrated associations between PM_2.5_ exposure and all-cause and cardiovascular mortality in the United States [3–8]. In addition to mortality, air pollution has also been associated with ischemic heart disease, stroke, and progression of atherosclerosis [3, 6, 9–12]. Associations between air pollution and cardiovascular disease have also been identified in other countries, including Europe and China. Long-term exposure to particulate matter was associated with acute coronary events in 11 European cohorts as part of the ESCAPE Project [13], and particulate matter and nitrogen dioxide were associated with increased risk of cardiovascular and cerebrovascular disease [14, 15]. In a recent review and meta-analysis, a 10 μg/m^3^ higher PM_2.5_ exposure was associated with a 6% (95% confidence interval [CI]: 4%, 8%) higher risk for all-cause and 11% (95% CI: 5%, 16%) higher risk for cardiovascular mortality [16]. Although much uncertainty exists regarding underlying biological mechanisms for this association, DNA methylation has been postulated to mediate the effects of air pollution on cardiovascular disease (CVD) [17].

DNA methylation is a chemical modification of DNA that involves the addition of a methyl group to cytosine and predominantly occurs at cytosine-guanine dinucleotide (CpG) sites [18]. DNA methylation in CpG-rich promoters tends to repress transcription whereas the effect is context-dependent in other genomic regions.

Previous studies show that DNA methylation patterns are associated with atherosclerosis [19], ischemic heart disease [20], and blood pressure while others have suggested DNA methylation as a biomarker for cardiovascular disease [21]. Moreover, exposure to air pollutants such as black carbon, particulate matter, benzene, and SO_4_ led to a genome-wide reduction in DNA methylation, or global hypomethylation, and were associated with methylation levels of candidate genes [22–26].

Results from past studies, however, are difficult to interpret due to several limitations. First, they were limited by the use of peripheral blood leukocytes, which are composed of a mixture of cells that have unique DNA methylation profiles. Since DNA methylation is cell-specific, it is more relevant to study purified samples of a single cell type such as monocytes. Monocytes play an important role in atherogenesis by promoting chronic inflammation and differentiating into macrophages that accumulate in plaques [27]. In addition, most prior studies did not measure gene expression to investigate the potential biological relevance of methylation differences. Some studies were further limited by potential exposure misclassification and measurement error in air pollution exposures.

We evaluated the association between long-term exposure to ambient air pollution and global and candidate CpG site DNA methylation in circulating monocytes of 1,207 participants from the Multi-Ethnic Study of Atherosclerosis (MESA). Candidate CpG sites were those with methylation previously associated with expression of nearby genes in the MESA cohort. For significant air pollution-associated methylation sites, we also assessed the association between expression of genes previously associated with the methylation sites and air pollution. MESA uniquely couples methylomic and transcriptomic data in monocytes with advanced air pollution exposure predictions.

## Methods

### Study population

MESA enrolled 6,814 participants aged 45–84 free of CVD at baseline from July 2000 to July 2002 at six field centers (Baltimore, MD; Chicago, Illinois; Los Angeles, CA; New York, NY; St. Paul, MN, and Winston-Salem, NC) with ongoing follow-up [28]. The MESA cohort is a diverse, population-based sample of 38% white, 28% black, 22% Hispanic, and 12% Asian participants, of whom half are women. Study participants underwent extensive physical exams to determine subclinical CVD and questionnaires to obtain information on sociodemographic, lifestyle, and psychosocial factors. MESA Air began in 2004 and builds on the original MESA study by adding new participants, outcome measurements, and air pollution exposure assessments of ambient fine particulate matter (PM_2.5_), oxides of nitrogen (NO_X_), and black carbon [29]. The MESA Epigenomics and Transcriptomics Study obtained genome-wide methylomic and transcriptomic profiles of CD14+ purified monocytes from 1,264 randomly selected MESA participants from four MESA field centers (Baltimore, MD; New York, NY; St. Paul, MN; and Winston-Salem, NC) at the 5th examination (April 2010-February 2012) [30]. The study protocol was approved by the Institutional Review Boards at Johns Hopkins Medical Institutions, University of Minnesota, Columbia University Medical Center, Wake Forest University Health Sciences, and University of Washington. All participants signed informed consent.

This study was restricted to participants who had available methylomic, transcriptomic, and air pollution data (*n* = 1,207). Since DNA methylation and gene expression data were not available for participants from the Los Angeles and Chicago MESA sites, there were no Asian participants in our analytic sample. Those included in the study were generally representative of all participants from the 5th examination at the four MESA field centers represented except for having fewer black participants, more Hispanic participants, and fewer participants from Winston-Salem, NC (see Additional file 1: Table S1).

### Air pollution assessment

PM_2.5_ and NO_X_ predictions are available through MESA Air as likelihood-based 2-week averages for all participants from 1999 to 2012. The air pollution prediction method has been described previously [31, 32]. Sources of monitoring data included the U.S. Environmental Protection Agency-operated Air Quality System monitors, including data from the Interagency Monitoring of Protected Visual Environments (IMPROVE) network, and cohort-specific monitoring. Additional data from the New York City Community Air Survey (NYCCAS) were used in New York City [32, 33]. In each study region, spatiotemporal models that accommodated unbalanced monitoring data were developed to predict ambient air pollution concentrations. The prediction model included monitoring data and over 300 geographic variables, such as distance to nearest road, population density, land use, and dispersion model outputs [31, 32]. Partial least squares was used to reduce the dimensionality of geographic covariates for inclusion in the models. Spatial smoothing was used to borrow strength between spatially close observations [32]. This modeling technique used the combined data to characterize seasonal and shorter-term time trends, key sources of spatial variability within the study communities, and underlying spatial and spatiotemporal correlation. For the present analyses, individual-level outdoor residential concentrations of PM_2.5_ and NO_X_ were averaged over the 12 months prior to blood draw.

### DNA methylation and gene expression quantification

Blood was drawn at the 5th examination (April 2010–February 2012). Monocytes were purified on-site by trained technicians following standardized protocols with extensive quality control measures using anti-CD14-coated magnetic beads and AutoMACs automated magnetic separation unit (Miltenyi Biotec, Bergisch Gladbach, Germany) [30]. DNA and RNA were extracted simultaneously from purified monocytes using AllPrep DNA/RNA Mini Kit (Qiagen, Inc., Hilden, Germany).

Genome-wide methylation profiles of over 485,000 CpG sites were characterized in purified monocytes using the Infinium HumanMethylation450 BeadChip (450k; Illumina, Inc. CA, USA). Bisulfite conversion of DNA fragments was performed with the EZ-96 DNA Methylation Kit (Zymo Research, Orange, CA, USA). Bead-level methylation data were summarized using the Illumina *GenomeStudio* software, and then raw methylation calls were normalized and converted to M-values (log ratio of methylated to unmethylated intensities). The M-value was used in statistical analyses due to better statistical performance in differential methylation analyses [34]. The Illumina HumanHT-12 v4 Expression BeadChip and Ilumina Bead Array Reader were used to obtain genome-wide expression profiles of over 48,000 transcripts (Illumina, Inc. CA, USA) [30]. A random sampling technique was used to assign samples, including control samples, to chips and positions to mitigate batch effects for both arrays. Data pre-processing using Bioconductor [35] in R [36] and quality control methods were previously described [30], and more details can be found in the Additional file 1. All allosome CpGs were removed prior to analysis.

To reduce dimensions of our genome-wide methylation data, we focused on CpG sites more likely to be functionally relevant. Previous work in this cohort identified 11,203 methylation sites that were associated with the *cis*-expression of 3,093 gene transcripts in a sample of 1,264 randomly selected MESA participants [30]. There were 2,713 unique CpG sites among the most significant CpG sites associated with each of the 3,093 transcripts. We tested the association of these 2,713 expression-associated methylation sites (eMS) with PM_2.5_ and NO_X_. In our study, eMS were annotated with respect to the gene with expression most significantly associated to the eMS. Additional nearest gene annotation for the top eMS can be found in the Additional file 1: Tables S2 and S3.

Alu and long interspersed nuclear elements (LINE)-1 repetitive element DNA methylation were used as surrogates for global DNA methylation. By intersecting probe locations from the Infinium 450k array with RepeatMasker [37], we identified 12,456 and 9,507 probes for Alu and LINE-1 repetitive elements, respectively, that passed quality control exclusion. Median Alu and LINE-1 DNA methylation were calculated for each participant for use in regression analyses.

### Statistical analysis

The associations between air pollution (PM_2.5_ or NO_X_) and methylation M-values at candidate CpGs were assessed with least squares regression and robust empirical Bayes moderated t-statistics using the *limma* package from Bioconductor [38]. To account for multiple testing of the 2,713 eMS, we controlled the false discovery rate (FDR) at 0.05 using the Benjamini and Hochberg method [39]. For eMS that were significantly associated with PM_2.5_ or NO_X_, we also tested the association between the transcript paired to that eMS and PM_2.5_ or NO_X_ using linear models with least squares regression and robust empirical Bayes moderated t-statistics using *limma*. The associations between air pollution and median Alu and median LINE-1 methylation were examined using linear regression with robust standard errors.

In all analyses, we adjusted for age, sex, race/ethnicity (black, Hispanic, white), household income, education, neighborhood socioeconomic status, smoking (smoking status and pack-years), secondhand smoke, body mass index, recent infection, methyl nutrient intake (continuous folate, vitamin B12, vitamin B6, methionine, zinc), physical activity, study site, microarray chip, and chip position (DNA methylation analysis only). We also adjusted for residual sample contamination by non-monocytes by adjusting for enrichment scores for neutrophils, B cells, T cells, and natural killer cells.

No covariate had more than 4% missing data. Multivariate imputation using chained equations was used to impute missing values in Stata 13 [40]. Since the proportion of missing data was low, only one iteration was used in association analyses.

Sensitivity analyses were conducted to investigate whether the association between exposure to PM_2.5_ and NO_X_ and Alu and LINE-1 methylation would be sensitive to a shorter averaging period of two weeks for the MESA Air likelihood-based air pollution predictions. In addition to including all covariates in the main study, this analysis also adjusted for temperature, relative humidity, month of blood draw, and day of week of blood draw. Additional sensitivity analyses were also conducted to assess potential effect modification by sex and race/ethnicity by fitting interaction terms with air pollution.

### Functional annotation analysis

*In silico* functional prediction of chromatin states in monocytes was performed using ChromHMM [41] to predict segmentation among six states, based on histone modifications in monocyte samples from the BLUEPRINT [42, 43] (H3K27ac, H3K4me1, H3K4me3) and the Encyclopedia of DNA Elements (ENCODE) [44] (H3K36me3) projects. Annotation also included DNase hypersensitive hotspot data in a monocyte sample (Sample ID RO01746, data generated by the UW ENCODE group) and transcription factor binding sites detected in any cell type available from ENCODE [44]. Data was accessed from the UCSC Genome Browser [45] and the Gene Expression Omnibus (https://www.ncbi.nlm.nih.gov/geo/).

## Results

**Fig. 1:**
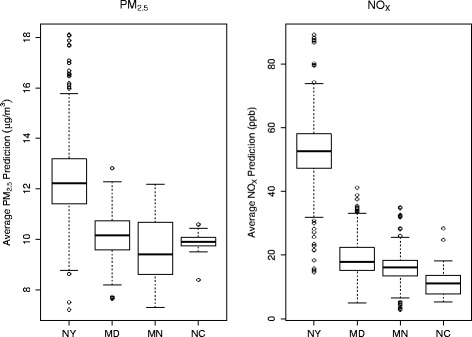
Ambient predictions of PM_2.5_ and NO_X_ by site, averaged over 12 months prior to blood draw. Site abbreviations: NY, New York; MD, Maryland; MN, Minnesota; NC, North Carolina. The lower and upper ends of the box represent the 25th and 75th percentiles, respectively, and the center bar represents the median. The bottom and top whiskers represent 1.5 times the interquartile distance above and below the 25th and 75th percentiles, respectively, and the circles indicate outliers

**Fig. 2:**
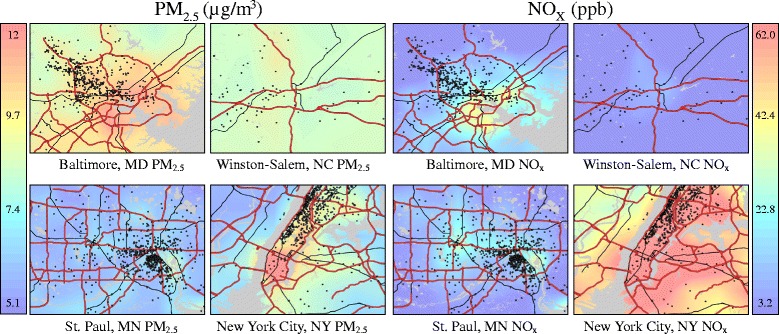
Maps of estimated PM_2.5_ and NO_X_ concentrations by MESA Air study site. The maps show smoothed air pollution predictions, which may reduce the visibility of fine-scale variation in concentrations. Black dots represent participant locations and are jittered to protect participant confidentiality

The analytic sample consisted of 1,207 participants with mean age of 69.6 (Table 1). Of these participants, 21.2% were black, 31.6% were Hispanic, and 47.2% were white. Roughly half were female, and most were former (50.3%) or never (40.3%) smokers with average body mass index of 29.7 kg/m^2^. Over 65% of participants received education beyond high school, and nearly 40% had income over $50,000. Median Alu and median LINE-1 methylation were 2.4 and 2.6 M-value units, respectively. Figure 1 shows plots of site-specific 12-month average ambient PM_2.5_ and NO_X_ predictions. The overall average PM_2.5_ and NO_X_ predictions were 10.7 μg/m^3^ (interquartile range [IQR] = 2.2 μg/m^3^) and 28.7 ppb (IQR = 31.9 ppb), respectively. PM_2.5_ and NO_X_ were positively correlated (correlation coefficient = 0.82). Figure 2 shows maps of PM_2.5_ and NO_X_ predictions over the four study regions, along with participant locations shown in black dots that are jittered to protect confidentiality.

**Table 1:** Descriptive characteristics of 1,207 MESA participants

	Total	New York	Maryland	Minnesota	North Carolina
	n (%) or mean ± SD	n (%) or mean ± SD	n (%) or mean ± SD	n (%) or mean ± SD	n (%) or mean ± SD
Age (y)	69.6 ± 9.4	69.7 ± 9.7	70.7 ± 9.0	68.5 ± 9.5	71.1 ± 7.0
Race/ethnicity, %					
White	570 (47.2)	82 (20.6)	173 (57.5)	267 (58.4)	48 (94.1)
Black	256 (21.2)	125 (31.4)	128 (42.5)	0 (0)	3 (5.9)
Hispanic	381 (31.6)	191 (48.0)	0 (0)	190 (41.6)	0 (0)
Sex, %					
Female	623 (51.6)	231 (58.0)	156 (51.8)	208 (45.5)	28 (54.9)
Male	584 (48.4)	167 (42.0)	145 (48.2)	249 (54.5)	23 (45.1)
Smoking status, %^a^					
Never	484 (40.3)	181 (45.8)	120 (40.1)	165 (36.3)	18 (35.3)
Former	604 (50.3)	184 (46.6)	149 (49.8)	244 (53.6)	27 (52.9)
Current	112 (9.3)	30 (7.6)	30 (10.0)	46 (10.1)	6 (11.8)
Secondhand smoke, (hours per week)^a^	3.7 ± 20.5	2.3 ± (11.1)	5.9 ± 35.3	3.3 ± 12.3	5.5 ± 14.2
Body mass index (kg/m^2^)^a^	29.7 ± 5.5	29.3 ± 5.6	30.1 ± 5.7	29.8 ± 5.3	28.5 ± 5.8
Physical activity (MET-min/wk m-su)^a^	5,696.2 ± 7,205.6	5,364.6 ± 7,139.0	5,978.8 ± 9,405.6	5,942.0 ± 5,770.6	4,405.0 ± 3,314.3
Education, %^a^					
Less than high school	176 (14.6)	94 (23.6)	20 (6.7)	61 (13.3)	1 (2.0)
High school	236 (19.6)	76 (19.1)	59 (19.7)	95 (20.8)	6 (11.8)
Some college but no degree	212 (17.6)	68 (17.1)	59 (19.7)	75 (16.4)	10 (19.6)
Bachelor’s/Associate/Technical	373 (31.0)	99 (24.9)	81 (27.1)	171 (37.4)	22 (43.1)
Advanced degree	208 (17.3)	61 (15.3)	80 (26.8)	55 (12.0)	12 (23.5)
Income, %^a^					
<$25,000	297 (25.5)	131 (33.5)	49 (17.3)	110 (24.9)	7 (14.3)
$25,000–$49,999	371 (31.9)	125 (32.0)	77 (27.2)	150 (34.0)	19 (38.8)
$50,000–$99,999	333 (28.6)	89 (22.8)	104 (36.7)	125 (28.3)	15 (30.6)
$100,000 or more	163 (14.0)	46 (11.8)	53 (18.7)	56 (12.7)	8 (16.3)
Neighborhood socioeconomic status factor score^a^	−0.41 ± 1.09	−1.06 ± 1.42	−0.27 ± 0.85	0.004 ± 0.62	−0.21 ± 0.91
Recent infection, %^a^					
No	922 (77.2)	293 (73.8)	225 (76.0)	365 (80.8)	39 (78.0)
Yes	273 (22.8)	104 (26.2)	71 (24.0)	87 (19.2)	11 (22.0)
Folate (mcg)^a^	317.1 ± 169.2	308.5 ± 162.7	301.9 ± 165.0	333.6 ± 175.6	324.1 ± 175.9
Vitamin B12 (mcg)^a^	3.9 ± 3.8	4.1 ± 5.1	3.6 ± 3.2	4.0 ± 2.8	3.9 ± 2.8
Vitamin B6 (mg)^a^	1.5 ± 0.8	1.4 ± 0.7	1.5 ± 0.9	1.6 ± 0.8	1.5 ± 0.7
Methionine (g)^a^	1.4 ± 0.8	1.3 ± 0.8	1.3 ± 0.9	1.5 ± 0.8	1.4 ± 0.8
Zinc (mg)^a^	8.9 ± 5.2	8.4 ± 4.9	8.3 ± 5.6	9.6 ± 5.2	8.8 ± 4.3

^a^ Contains missing values

**Fig. 3:**
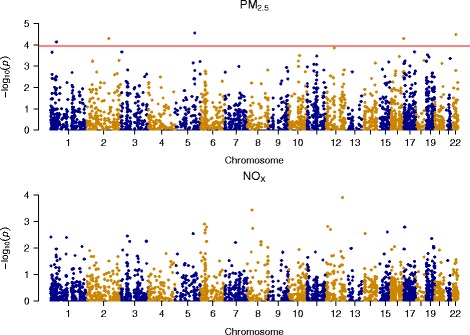
Association between PM_2.5_ and NO_X_ and 2,713 expression-associated methylation sites. Red line demarcates statistical significance at a false discovery rate of 5%. Plot for NO_X_ does not have a red line because no sites were statistically significant

Figure 3 shows a plot of the –log_10_(p-values) against genomic position from linear regression analysis of 2,713 eMS with PM_2.5_. We found five eMS had methylation significantly (FDR <0.05) associated with PM_2.5_ (four positively and one negatively), and they included cg20455854 (*ANKHD1*; ankyrin repeat and KH domain containing 1), cg07855639 (*LGALS2*; lectin, galactoside-binding, soluble, 2), cg07598385 (*ANKRD11*; ankyrin repeat domain 11), cg17360854 (*BAZ2B*; bromodomain adjacent to zinc finger domain, 2B), and cg17360854 (*PPIE*; peptidylprolyl isomerase E). The significant eMS associated with PM_2.5_ are shown in Table 2. The eMS cg20455854 has methylation associated with *ANKHD1* expression and is the eMS with the smallest p-value for the association with PM_2.5_ (coefficient = 0.139, 95% CI: 0.074, 0.203; per 2.5 μg/m^3^; *p* = 2.77 × 10^−5^). Cg20455854 is located within a DNase hypersensitivity site (monocyte data from ENCODE), a transcription factor binding site (any cell type from UCSC Genome Browser), and a predicted strong enhancer region (see Additional file 1: Table S2). NO_X_ exposure was not significantly associated with methylation of any eMS (see Additional file 1: Table S3).

**Table 2:** eMS with PM_2.5_-associated methylation and association between corresponding transcript gene expression with PM_2.5_, ranked by gene expression association with PM_2.5_

		DNA Methylation				Gene Expression			
Gene^a^	Chr	CpG	β (95% CI)	*P*-value	Adjusted *P*-value	Illumina Transcript	β (95% CI)	*P*-value	Adjusted *P*-value
PM_2.5_									
*ANKHD1*	5	cg20455854	0.139 (0.074, 0.203)	2.77 × 10^−5^	0.034	ILMN_1765091	−0.048 (−0.074, −0.022)	3.71 × 10^−4^	0.002
*LGALS2*	22	cg07855639	0.081 (0.043, 0.120)	3.28 × 10^−5^	0.034	ILMN_1687306	−0.147 (−0.240, −0.053)	0.002	0.005
*ANKRD11*	16	cg07598385	0.108 (0.056, 0.160)	4.97 × 10^−5^	0.034	ILMN_2108709	−0.075 (−0.142, −0.008)	0.028	0.046
*BAZ2B*	2	cg17360854	0.081 (0.042, 0.120)	5.08 × 10^−5^	0.034	ILMN_1720850	−0.016 (−0.060, 0.027)	0.463	0.578
*PPIE*	1	cg23599683	−0.057 (−0.085, −0.029)	7.17 × 10^−5^	0.039	ILMN_1680341	0.004 (−0.020, 0.028)	0.728	0.728

PM_2.5_, fine particulate matter (per 2.5 μg/m^3^); *chr* chromosome, *CI* confidence interval

^a^ Gene corresponding to Illumina transcript associated with CpG methylation

Although all candidate CpGs had methylation previously associated with mRNA expression of a nearby gene in this cohort, not all of these mRNA expression profiles associated with PM_2.5_-associated eMS were significantly (FDR <0.05) differentially expressed with respect to air pollution. Three of the five genes with mRNA expression previously associated with the significant PM_2.5_-eMS had mRNA expression also associated with PM_2.5_. PM_2.5_ exposure was negatively associated with *ANKHD1* (coefficient = −0.048; 95% CI: −0.074, −0.022; per 2.5 μg/m^3^; *p* = 3.71 × 10^−4^), *LGASL2* (β = −0.147; 95% CI: −0.240, −0.053; per 2.5 μg/m^3^; *p* = 0.002), and *ANKRD11* expression (β = −0.075; 95% CI: −0.142, −0.008; per 2.5 μg/m^3^; *p* = 0.028) (Table 2).

Median DNA methylation of Alu was not associated with PM_2.5_ (coefficient = −0.003; 95% CI: −0.006, 0.001; *p* = 0.155) or NO_X_ (coefficient = 0.001; 95% CI: −0.006, 0.008; *p* = 0.719) after covariate adjustment (Table 3). Median DNA methylation of LINE-1 was also not associated with PM_2.5_ (coefficient = −0.003; 95% CI: −0.007, 0.001; *p* = 0.177) or NO_X_ (coefficient = −0.0004; 95% CI: −0.009, 0.008; *p* = 0.927) after covariate adjustment (Table 3). Results were not sensitive to using a 2-week averaging period for air pollutants (see Additional file 1: Table S4). We did not identify interactions between air pollution and sex or race/ethnicity (data not shown).

**Table 3:** Association between global DNA methylation (Alu and LINE-1) and one-year average PM_2.5_ (per 2.5 μg/m^3^) and NO_X_ (per 30 ppb)

	PM_2.5_		NO_X_	
	β (95% CI)^a^	*P*-value	β (95% CI)^a^	*P*-value
Alu	−0.003 (−0.006, 0.001)	0.155	0.001 (−0.006, 0.008)	0.719
LINE-1	−0.003 (−0.007, 0.001)	0.177	−0.0004 (−0.009, 0.008)	0.927

PM_2.5_, fine particulate matter; *NOX* oxides of nitrogen, *CI* confidence interval, *LINE-1* long interspersed element 1

^a^ Models adjusted for age, race/ethnicity, sex, study site, income, education, neighborhood socioeconomic status factor score, cigarette smoking, secondhand smoke, body mass index, physical activity, methyl nutrient intake (folate, vitamin B12, vitamin B6, methionine, zinc), residual cell contamination by non-monocytes, recent infection, and methylation chip position. Methylation values were adjusted for methylation chip prior to regression analysis

## Discussion

We examined the associations between long-term air pollution exposure and DNA methylation in monocytes. We found air pollution to be significantly associated with site-specific DNA methylation, but not global DNA methylation. In order to identify potentially functionally relevant genes involved in the pathogenesis of air pollution-related cardiovascular disease, we focused on a set of CpG sites that were previously associated with expression of nearby genes. In monocytes, we detected five CpGs with methylation associated with long-term PM_2.5_ exposure, of which three were potentially functionally related to genes with expression also correlated with air pollution. No site-specific methylation sites were associated with long-term NO_X_ exposure.

Genes with both regulatory DNA methylation site(s) and mRNA expression that are associated with air pollution may be more plausibly involved in the pathogenesis of air pollution-related disease. In this study, we identified three such genes: *ANKHD1*, *LGALS2*, and *ANKRD11*. While we did not formally evaluate whether DNA methylation mediates the association of air pollution with atherosclerotic cardiovascular disease in this report and temporal relationships are not clear, the identification of these genes suggests that DNA methylation may potentially link air pollution inhalation with changes in monocyte gene expression which could contribute to disease.

ANKHD1 function in monocytes is unknown, but it is highly expressed in acute leukemia, multiple myeloma, and prostate cancer and may support proliferation and cell cycle progression of cancer cells [46–49]. ANKHD1 contains multiple ankyrin repeats [50], which mediate protein-protein interactions and have been found in proteins involved in various cellular functions including cell-cell signaling, cytoskeleton integrity, cell cycle control, transcriptional regulation, and inflammatory response [51]. The role that ANKHD1 plays in air pollution-related disease is unclear, but the presence of ankyrin repeats suggests that ANKHD1 may play an important cellular function. This study is the first to demonstrate an association between an environmental pollutant and *ANKHD1* DNA methylation and expression. In our study, PM_2.5_ exposure correlated with higher methylation of *ANKHD1*-eMS cg20455854 and lower mRNA expression of *ANKHD1*. The location of cg20455854 within a DNase hypersensitivity site, transcription factor binding site, and predicted strong enhancer region provides supports a potentially functional PM_2.5_-eMS.

*LGALS2* encodes the protein galectin-2, which is part of the galectin family [52]. Prior studies of LGALS2 function and disease associations have been inconsistent and sometimes conflicting. Higher galectin-2 levels in monocytes and macrophages are linked to low arteriogenic response in coronary artery disease patients [53] and inflammation [54]. A case–control study in Japan first reported that the 3279C > T(rs7291467) polymorphism, which reduces galectin expression, was negatively associated with risk of myocardial infarction [55]. This single nucleotide polymorphism is located over 380 kb away from the *LGALS2*-eMS cg07855639. However, subsequent studies in other Asian and European populations provided mixed results [56–61]. Studies even found the TT genotype, which leads to lower levels of galectin-2, to be associated with more severe coronary stenosis [62] and higher levels of C-reactive protein [60]. Moreover, addition of galectin-2 to activated T cells downregulates production of the pro-inflammatory cytokines interferon-γ and tumor necrosis factor-α, while increasing secretion of interleukin-5 and the anti-inflammatory cytokine interleukin-10 [63].

Thus while prior studies suggest that galectin-2 may play a role in cardiovascular disease, potentially by modulating both pro-inflammatory and anti-inflammatory cytokines, the mechanism and direction of association remain to be determined. In our study, PM_2.5_ exposure was positively associated with methylation of the *LGALS2*-eMS cg07855639 and negatively associated with *LGALS2* mRNA expression in monocytes. While these results suggest that air pollution may potentially affect inflammation, it is difficult to interpret our results in the context of past studies given the inconsistency in galectin-2 disease associations.

*ANKRD11* regulates chromatin modification and has been found to be associated with autism [64] and KBG syndrome (rare disorder marked by dental, neurobehavioral, craniofacial and skeletal anomalies) [65]. In our cohort, PM_2.5_ was positively associated with *ANKRD11* methylation and negatively associated with *ANKRD11* mRNA expression. Although air pollution is a risk factor for cardiovascular disease, air pollution has also been linked to other complex diseases such as autism, where the common mechanism might be inflammation [66].

*BAZ2B* and *PPIE* methylation, but not mRNA expression, were significantly associated with PM_2.5_ exposure. *BAZ2B* encodes a bromodomain containing chromatin remodeling protein that epigenetically regulates transcription. Although the function of *BAZ2B* remains unclear, single nucleotide polymorphisms in *BAZ2B* were associated with sudden cardiac death [67]. PPIE belongs to the family of PPIases with proline isomerase activity that stimulates folding and conformational changes in proteins and may be linked to leukemia [68], colorectal cancer [69], and body mass index [70]. Although it is uncertain how these genes may be related to air pollution-related disease, this is the first study to report the novel associations between air pollution and *BAZ2B* and *PPIE*.

While many prior studies focused on the association between air pollution exposure and global DNA methylation, there is also evidence that air pollution is associated with DNA methylation of specific genes. Previous studies identified significant associations between air pollution exposure and differences in DNA methylation of several genes including *iNOS* [71, 72], tissue factor, intercellular adhesion molecule 1, toll-like receptor 2, interferon-γ, and interleukin-6 [23]. In addition, an epigenome-wide study of whole blood using the 450k array identified 12 CpG sites associated with short- and mid-term PM_2.5_ exposures at genome-wide significance [73]. Of these genes and CpG sites with methylation previously reported to be associated with air pollution, we only analyzed methylation of intercellular adhesion molecule 1 in this study, and it was not associated with long-term PM_2.5_ or NO_X_ exposure. The other genes were not represented among our list of candidate methylation sites, so we did not analyze the association between their DNA methylation and air pollution exposure.

We were unable to replicate prior observational studies that found significant associations between air pollution exposure and global DNA methylation using Alu and LINE-1 methylation as surrogates [22, 26, 74]. Short-term controlled human experiments (blood collected 1–30 h post-exposure) showed that exposure to air pollutants changed DNA methylation levels of Alu and LINE-1 elements [75, 76]. However, other studies did not find an association between air pollution and LINE-1 and/or Alu DNA methylation [22, 71, 74].

Differences in assay method may partially explain our null associations with global DNA methylation. We assessed global DNA methylation using probes on the Illumina 450k array that map to Alu and LINE-1 repetitive elements, unlike most previous studies that used pyrosequencing. We also adjusted for a wider array of potential confounders not available in prior studies. Furthermore, our study examined DNA methylation in purified monocytes whereas previous studies used a mixture of blood cells. Monocytes constitute about 5-10% of peripheral blood leukocytes [77], highlighting the possibility that the association between air pollution and global DNA hypomethylation may be specific to other leukocytes. Moreover, our study population differs from those of prior studies. Our study included men and women age 65 and over who reside in six U.S. regions. Prior studies included a variety of study populations including an elderly cohort of men from the Boston area (mean age 73), [22, 25] healthy male workers from an electric steel furnace plant in Italy (mean age 44), [26] and a predominantly male group of workers from the Ma Ta Phut industrial estate in Thailand (mean age 31) [74].

We considered the possibility that the difference in air pollution exposure time windows may explain our non-significant findings. Previous studies found significant associations for short- and intermediate-term air pollution exposure, rather than long-term exposures that are the primary focus of the MESA Air project. A study of air pollution and percent 5-methyl-2′-deoxycytidine methylation found the strongest associations above 7 days, with most of the effects observed for the 5- to 30-day moving average exposures [78]. However, in our study, the associations between 2-week average air pollutant concentrations and global DNA methylation were not significant in sensitivity analyses.

In our study, we only identified CpGs significantly associated with PM_2.5_ but not NO_X_ (at FDR of 0.05). Sources of ambient PM_2.5_ include energy generation, combustion of fuel, agricultural and industrial processes, and road and wind-blown dust [79]. While motor vehicle emissions contribute to ambient PM_2.5_, these emissions are one of the main anthropogenic sources of ambient NO_X_. Thus, differences in sources and composition of these two pollutants may partially explain why observed associations of PM_2.5_ with methylation were not observed for NO_X_.

There are limitations to our study. Our analysis was cross-sectional, and we were unable to make inferences about the effect of air pollution on changes in DNA methylation. In addition, our study was comprised of individuals in the United States, which has relatively low air pollution concentrations compared to many parts of the world. Higher concentrations of air pollutants may trigger alternate disease mechanisms not captured in this analysis. Additionally, there may be effect modifiers of the air pollution-DNA methylation association not investigated in this study. However, in our sensitivity analyses we did not find evidence of effect modification by sex or race/ethnicity. Finally, there may be residual confounding by factors not included in the analysis.

Our study is distinct in several ways. Our focus on purified monocytes greatly enhances the interpretability of our results and our ability to tease out monocyte-specific differentially methylated signals that are important in atherosclerosis. Furthermore, the population-based sampling, younger age, and multiracial nature of the MESA cohort allows for greater generalizability of study results. The coupling of DNA methylation and gene expression data aids the detection of differentially methylated sites that may be functionally relevant. Finally, the state-of-art air pollution assessment in MESA Air provides individual-level exposures that capture fine-scale spatial variability in air pollution to greatly reduce measurement error.

## Conclusions

Long-term ambient air pollution exposure was associated with site-specific DNA methylation, but not global DNA methylation, in purified blood monocytes obtained from a multi-ethnic adult population. We identified genes that may be functionally relevant to mediating air pollution health effects by focusing on expression-associated methylation sites. We report novel associations between PM_2.5_ exposure and DNA methylation and expression of *ANKHD1*, *LGALS2*, and *ANKRD11*. Future research in blood monocytes could address whether air pollution-associated diseases—such as atherosclerosis—may be mediated in part by DNA methylation epigenetic reprogramming.

## Additional file

Additional file 1: Supplemental Material. (DOCX 39 kb)

## Abbreviations

450k: HumanMethylation450 BeadChip
*ANKHD1*: Ankyrin repeat and KH domain containing 1
*ANKRD11*: Ankyrin repeat domain 11
*BAZ2B*: Bromodomain adjacent to zinc finger domain, 2B
CI: Confidence interval
CpG site: Cytosine-guanine dinucleotide site
CVD: Cardiovascular disease
eMS: Expression-associated methylation sites
ENCODE: Encyclopedia of DNA Elements
FDR: False discovery rate
IQR: Interquartile range
*LGALS2*: Lectin, galactoside-binding, soluble, 2
LINE: Long interspersed nuclear elements
MESA: Multi-Ethnic Study of Atherosclerosis
NO_X_: Oxides of nitrogen
PM_2.5_: Fine particulate matter
*PPIE*: Peptidylprolyl isomerase E
