# Supplementary material for: Long-term outdoor air pollution and DNA methylation in circulating monocytes: results from the Multi-Ethnic Study of Atherosclerosis (MESA)
Source: Environ Health. 2016 Dec 1;15:119. doi: 10.1186/s12940-016-0202-4 (PMC5131503; doi:10.1186/s12940-016-0202-4)
Supplement: Additional file 1: — Supplemental Material. (DOCX 39 kb) [file 12940_2016_202_MOESM1_ESM.docx]

**Supplemental Material**

**Long-term outdoor air pollution and DNA methylation in circulating monocytes: Results from the Multi-Ethnic Study of Atherosclerosis (MESA)**

Gloria C. Chi, Yongmei Liu, James W. MacDonald, R. Graham Barr, Kathleen M. Donohue, Mark D. Hensley, Lifang Hou, Charles E. McCall, Lindsay M. Reynolds, David S. Siscovick, Joel D. Kaufman

**Table of Contents**

Supplemental Text, pages 2-4

Table S1, page 5

Table S2, page 7

Table S3, page 8

Table S4, page 9

**Supplemental Text**

*Quality Control and Microarray Pre-processing*

Monocytes samples were consistently >90% pure based on flow cytometry of 18 specimens. A NanoDrop spectrophotometer measured optical density measurements for DNA and RNA quality control. The integrity of 18s and 28s ribosomal RNA was also evaluated using the Agilent 2100 Bioanalyzer with RNA 6000 Nano chips (Agilent Technology, Inc., Santa Clara, CA, USA), and RNA with RNA integrity (RIN) scores >9.0 were included in the expression microarrays.

Data pre-processing was performed using Bioconductor (Gentleman et al. 2004) in R (R Core Team 2015). For expression data, Illumina’s GenomeStudio was used to correct for local background and the *beadarray* package (Dunning et al. 2007) was used for quality check analyses and bead type summarization. Negative controls on the array were used to compute detection P-values. The *neqc* function of the *limma* package was used to perform a normal-exponential convolution model analysis to estimate non-negative signal, quantile normalization using all probes (gene and control, detected and not detected) and samples, addition of a recommended (small) offset, log2 transformation, and elimination of control probe data from the normalized expression matrix. Multidimensional scaling plots showed the five common control samples were highly clustered together and identified three outlier samples, which were excluded subsequently.

Normalization of the Illumina HumanMethylation450 Beadchip two-channel systems was performed by 1) smooth quantile normalization, 2) subtraction of the median intensity value of negative control probes, and 3) standard quantile normalization applied to bead-type intensities and combined across Infinium I and II assays and both colors, using the *lumi* package (Du et al. 2008). Quality control involved checking for sex and race/ethnicity mismatches and outlier identification by multidimensional scaling plots.

For quality control, we excluded probes designed for sequences on the X or the Y chromosome, uninformative probes (defined as those probes with a detection p-value > 0.05 in > 10% of subjects), and probes with single nucleotide polymorphisms within 10 bp.

A database of the 11,203 significant methylation-gene expression association results can be downloaded at <http://www.wakehealth.edu/Research/Public-Health-Sciences/MesaEpiGenomics/eMS-database---The-MESA-EpiGenomics-Project.htm>. All 2,713 candidate eMS are annotated with respect to their most significantly associated transcript from this eMS database.

*Adjustment Covariates*

Age, sex, race/ethnicity, household income, smoking, secondhand smoke, recent infection, and physical activity were collected from questionnaires administered at the 5^th^ exam. Education was obtained from a questionnaire administered at the 1^st^ exam. Food frequency questionnaire was used to calculate intake of methyl nutrients including folate, vitamin B12, vitamin B6, methionine, and zinc. Body mass index (kg/m^2^) was calculated from measured body weight and height at the 5^th^ exam. Gene set enrichment analysis of gene expression values for each participant was used to estimate enrichment scores for neutrophils, B cells, T cells, and natural killer cells based on the gene signature of each blood cell type from previously defined lists (Liu et al. 2013). The *ComBat* function in the package *SVA* (Leek et al. 2012) was used to obtain methylation chip-adjusted M-values for use in regression analyses.

**References**

Dunning MJ, Smith ML, Ritchie ME, Tavaré S. 2007. beadarray: R classes and methods for Illumina bead-based data. Bioinformatics 23:2183–2184; doi:10.1093/bioinformatics/btm311.

Du P, Kibbe WA, Lin SM. 2008. lumi: a pipeline for processing Illumina microarray. Bioinforma. Oxf. Engl. 24: 1547–1548.

Gentleman RC, Carey VJ, Bates DM, Bolstad B, Dettling M, Dudoit S, et al. 2004. Bioconductor: open software development for computational biology and bioinformatics. Genome Biol. 5: R80.

Leek JT, Johnson WE, Parker HS, Jaffe AE, Storey JD. 2012. The sva package for removing batch effects and other unwanted variation in high-throughput experiments. Bioinforma. Oxf. Engl. 28: 882–883.

Liu Y, Ding J, Reynolds LM, Lohman K, Register TC, Fuente ADL, et al. 2013. Methylomics of gene expression in human monocytes. Hum. Mol. Genet. 22: 5065–5074.

R Core Team. 2015. *R: A language and environment for statistical computing*. R Foundation for Statistical Computing, Vienna, Austria.

**Table S1.** Demographics of analytic sample and all participants from the 5^th^ examination from four Multi-Ethnic Study of Atherosclerosis study sites.

|  | Analytic Sample (n=1,207) | Exam 5 Participants^a^  (n=3,052) |
| --- | --- | --- |
|  | N (%) or Mean ± SD | N (%) or Mean ± SD |
| Age (y) | 69.6 ± 9.4 | 69.8 ± 9.4 |
| Race/ethnicity, % |  |  |
| Black | 256 (21.2) | 943 (30.9) |
| Chinese-American | 0 (0.0) | 2 (0.1) |
| Hispanic | 381 (31.6) | 699 (22.9) |
| White | 570 (47.2) | 1,408 (46.1) |
| Sex, % |  |  |
| Female | 623 (51.6) | 1,660 (54.4) |
| Male | 584 (48.4) | 1,392 (45.6) |
| Smoking status, %^b^ |  |  |
| Never | 484 (40.3) | 1,249 (41.5) |
| Former | 604 (50.3) | 1,493 (49.6) |
| Current | 112 (9.3) | 266 (8.8) |
| Secondhand smoke, (hours per week)^b^ | 3.7 ± 20.5 | 3.2 ± 16.8 |
| Body mass index (kg^2^/m^2^)^b^ | 29.7 ± 5.5 | 29.3 ± 5.7 |
| Physical activity (MET-min/wk m-su)^b^ | 5,696.2 ± 7205.6 | 5,458.2 ± 6566.0 |
| Education, %^b^ |  |  |
| Less than high school | 176 (14.6) | 402 (13.2) |
| High school | 236 (19.6) | 611 (20.1) |
| Some college but no degree | 212 (17.6) | 524 (17.2) |
| Bachelor's/Associate/Technical | 373 (31.0) | 962 (31.6) |
| Advanced degree | 208 (17.3) | 546 (17.9) |
| Income, %^b^ |  |  |
| < $24,999 | 297 (25.5) | 721 (25.0) |
| $25,000 - $49,999 | 371 (31.9) | 912 (31.6) |
| $50,000 - $99,999 | 333 (28.6) | 815 (28.2) |
| $100,000 or more | 163 (14.0) | 437 (15.1) |
| Neighborhood socioeconomic status factor score^b^ | -0.41 ± 1.1 | -0.34 ± 1.1 |
| Folate (mcg)^b^ | 317.1 ± 169.2 | 311.7 ± 214.7 |
| Vitamin B12 (mcg)^b^ | 3.9 ± 3.8 | 3.9 ± 5.5 |
| Vitamin B6 (mg)^b^ | 1.5 ± 0.8 | 1.5 ± 1.0 |
| Methionine (g)^b^ | 1.4 ± 0.8 | 1.4 ± 1.1 |
| Zinc (mg)^b^ | 8.9 ± 5.2 | 8.7 ± 6.3 |
| Recent infection, %^b^ |  |  |
| No | 922 (77.2) | 2,322 (77.3) |
| Yes | 273 (22.8) | 682 (22.7) |
| Study site, % |  |  |
| Winston-Salem, NC | 49 (4.1) | 813 (26.6) |
| New York, NY | 398 (33.0) | 810 (26.5) |
| Baltimore, MD | 303 (25.1) | 658 (21.6) |
| St. Paul, MN | 457 (37.9) | 771 (25.3) |
| PM_2.5_ (µg/m^3^)^b^ | 10.7 ± 1.7 | 10.7 ± 1.5 |
| NO_X_ (ppb)^b^ | 28.7 ± 18.5 | 24.8 ± 18.5 |

^a^ All participants at 5^th^ exam from four Multi-Ethnic Study of Atherosclerosis study sites: Baltimore, MD; New York, NY; St. Paul, MN; and Winston-Salem, NC.

^b^ Contains missing values.

**Table S2. Nearest gene and functional annotation of significant expression-associated methylation sites (eMS) associated with long-term PM_2.5_ exposure in the Multi-Ethnic Study of Atherosclerosis (n=1,207).**

| **CpG** | **Chr** | **Position (hg19)** | **Transcript Gene^a^** | **Illumina Transcript^b^** | **Nearest TSS Gene^c^** | **Distance TSS^d^** | **CD14+ Chromatin State ^e^** | **DNase HS CD14+^f^** | **TFBS Any Cell^g^** |
| --- | --- | --- | --- | --- | --- | --- | --- | --- | --- |
| cg20455854 | 5 | 139040849 | *ANKHD1* | ILMN_1765091 | *CXXC5* | 12330 | Strong enhancer | Yes | Yes |
| cg07855639 | 22 | 37959465 | *LGALS2* | ILMN_1687306 | *CDC42EP1* | 2993 | Heterochromatin | Yes | Yes |
| cg07598385 | 16 | 88566483 | *ANKRD11* | ILMN_2108709 | *MIR5189* | 31156 | Heterochromatin | No | Yes |
| cg17360854 | 2 | 161224771 | *BAZ2B* | ILMN_1720850 | *MIR4785* | 39620 | Strong enhancer | Yes | Yes |
| cg23599683 | 1 | 40235372 | *PPIE* | ILMN_1680341 | *OXCT2* | 1646 | Heterochromatin | No | No |

PM_2.5_, fine particulate matter; chr, chromosome; TSS, transcription start site; DNAse HS, DNase hypersensitivity; TFBS, transcription factor binding site; ENCODE, The Encyclopedia of DNA Elements.

^a^ All CpG sites have previously been associated with cis-gene expression. This annotation is based on gene that the associated transcript maps to.

^b^ Illumina transcript identification number for transcript associated with CpG.

^c^ Annotation based on gene with the nearest TSS.

^d^ Distance to nearest TSS.

^e^ Prediction based on histone modifications in monocyte samples from the BLUEPRINT (H3K27ac, H3K4me1, H3K4me3) and ENCODE (H3K36me3) projects.

^f^ DNase hypersensitivity reported in a CD14+ monocyte sample (ENCODE).

^g^ TFBS reported in any cell type available from the UCSC Genome Browser.

**Table S3. Top 5 expression-associated methylation sites (eMS) associations with NO_X_ (per 30 ppb) in the Multi-Ethnic Study of Atherosclerosis (n=1,207).**

| **CpG** | **Chr** | **Position** | **Transcript Gene^a^** | **Illumina Transcript^b^** | **Nearest TSS Gene^c^** | **Distance TSS^d^** | **β** | **95% CI** | **P-value** | **Adjusted P-value^e^** |
| --- | --- | --- | --- | --- | --- | --- | --- | --- | --- | --- |
| cg16859127 | 12 | 113897132 | OAS1 | ILMN_1658247 | LHX5 | 12743 | -0.201 | (-0.304, -0.099) | 1.27E-04 | 0.346 |
| cg16734451 | 8 | 32430022 | NRG1 | ILMN_1737252 | NRG1 | 23322 | 0.098 | (0.044, 0.152) | 3.70E-04 | 0.502 |
| cg22243996 | 6 | 26757644 | HIST1H2BD | ILMN_1758623 | ZNF322 | 97663 | 0.147 | (0.058, 0.236) | 1.27E-03 | 0.604 |
| cg03014680 | 12 | 10122522 | CLEC12A | ILMN_2292178 | CLEC12A | 1484 | 0.103 | (0.039, 0.167) | 1.55E-03 | 0.604 |
| cg01092361 | 6 | 42185687 | MRPS10 | ILMN_1663664 | MRPS10 | 53 | 0.115 | (0.043, 0.186) | 1.64E-03 | 0.604 |

NO_X_, oxides of nitrogen; chr, chromosome; TSS, transcription start site; CI, confidence interval.

^a^ All CpG sites have previously been associated with cis-gene expression. This annotation is based on gene that the associated transcript maps to.

^b^ Illumina transcript identification number for transcript associated with CpG.

^c^ Annotation based on gene with the nearest TSS.

^d^ Distance to nearest TSS.

^e^ Based on false discovery rate cutoff of 0.05. None of these eMS are statistically significantly associated with NO_X_ exposure at this false discovery rate cutoff.

**Table S4. Association between global DNA methylation (Alu and LINE-1) and 2-week average PM_2.5_ (per 2.5 µg/m^3^) and NO_X_ (per 30 ppb).**

|  | PM_2.5_ | | NO_X_ | |
| --- | --- | --- | --- | --- |
|  | β (95% CI) | P-value | β (95% CI) | P-value |
| Alu | 0.0004 (-0.002, 0.002) | 0.694 | 0.002 (-0.004, 0.008) | 0.464 |
| LINE-1 | 0.001 (-0.001, 0.003) | 0.415 | 0.003 (-0.004, 0.010) | 0.391 |

PM_2.5_, fine particulate matter; NO_X_, oxides of nitrogen; CI, confidence interval; LINE-1, long interspersed element. Models adjusted for age, race/ethnicity, sex, study site, income, education, neighborhood socioeconomic status factor score, cigarette smoking, secondhand smoke, body mass index, physical activity, methyl nutrient intake (folate, vitamin B12, vitamin B6, methionine, zinc), residual cell contamination by non-monocytes, recent infection, methylation chip position, temperature, relative humidity, month of blood draw, and day of week of blood draw. Methylation values were adjusted for methylation chip prior to regression analysis.
